# Spatial variability in the reproduction number of Ebola virus disease, Democratic Republic of the Congo, January–September 2019

**DOI:** 10.2807/1560-7917.ES.2019.24.42.1900588

**Published:** 2019-10-17

**Authors:** Kenji Mizumoto, Amna Tariq, Kimberlyn Roosa, Jun Kong, Ping Yan, Gerardo Chowell

**Affiliations:** 1 Graduate School of Advanced Integrated Studies in Human Survivability, Kyoto University Yoshida-Nakaadachi-cho, Sakyo-ku, Kyoto, Japan; 2 Hakubi Center for Advanced Research, Kyoto University, Yoshidahonmachi, Sakyo-ku, Kyoto, Japan; 3 Department of Population Health Sciences, School of Public Health, Georgia State University, Atlanta, Georgia, United States of America; 4 Department of Mathematics and Statistics, Georgia State University, Atlanta, Georgia, United States of America; 5 Department of Computer Science, Georgia State University, Atlanta, Georgia, United States of America; 6 Department of Computer Science, Emory University, Atlanta, Georgia, United States of America; 7 Public Health Agency of Canada, Ottawa, Canada; 8 Division of International Epidemiology and Population Studies, Fogarty International Center, National Institutes of Health, Bethesda, Maryland, United States of America

**Keywords:** Reproduction number, ebola, conflict, outbreak, next generation matrix, Congo, epidemic, threshold

## Abstract

The ongoing Ebola virus disease epidemic (August 2018─October 2019) in the Democratic Republic of the Congo, has been exacerbated by deliberate attacks on healthcare workers despite vaccination efforts. Using a mathematical/statistical modelling framework, we present the quantified effective reproduction number (*R*_t)_ at national and regional levels as at 29 September. The weekly trend in *R*_t_ displays fluctuations while our recent national-level *R*_t_ falls slightly above 1.0 with substantial uncertainty, which suggests improvements in epidemic control.

Since its emergence in 1976, Ebola virus disease (EVD) has caused multiple outbreaks in several African countries, including the Democratic Republic of the Congo (DRC) [1]. The current epidemic in DRC that emerged in August 2018 contrasts with previous Ebola outbreaks in that transmission chains have persisted for over a year in a region of conflict, despite the availability of a highly effective vaccine [2]. Here, we seek to characterise the transmission potential of EVD and generate short-term forecasts at the national and health zone (HZ) levels, focusing on the recent dynamics of the effective reproduction number (*R*_t_). We also discuss our transmission estimates considering changes in surveillance indicators and frequency of outbreaks of violence.

## Current situation in the Democratic Republic of the Congo

In terms of morbidity and mortality, the ongoing 2018–19 EVD outbreak in DRC was only surpassed in magnitude by the 2013–16 Western African epidemic [3]. Militia attacks and ethnic violence have been occurring in the affected region in DRC, which has fuelled a climate of community distrust of the government and of public health authorities resulting in fewer people seeking medical care for EVD [4,5]; healthcare centres and healthcare teams have been exclusively targeted undermining epidemiological surveillance efforts e.g. active case finding and isolation of infectious individuals, which are key for assessing the current state of the EVD epidemic and guiding public health efforts [5-7].

Ebola transmission hotspots in the DRC have varied geographically through the course of the outbreak [8]. At the beginning, the primary disease hotspots were centred in the HZ of Beni (HZ 3), Mabalako (HZ 1), Mandima (HZ 2), Butembo and Masereka HZ [5]. By December 2018, Katwa and Komanda HZs also exhibited intensified Ebola transmission [5]. The location of Ebola hotspots has been correlated with the frequency and location of outbreaks of violence and protests from community members that hinder the Ebola response efforts [5,9,10].

## Epidemiological incidence cases

Incidence curves of confirmed and probable cases of the ongoing Ebola epidemic in the DRC (August 2018–October 2019) at the national and HZ levels are publicly available in weekly reports on the World Health Organization (WHO) website [5,9]. The latest national Ebola incidence curve by date of symptoms onset, was published on 8 October 2019 in Situation Report 62 [5]. At the HZ level, incidence curves were retrieved from the WHO Disease Outbreak News Report published on 10 October 2019 [9]. The date of reporting for the national incidence curve was defined as 6 October 2019 and the date of reporting for the HZ incidence curves was defined as 10 October 2019. Week of symptoms onset and week of reporting for each new Ebola case were obtained by analysing consecutive Ebola WHO reports (Situation Report 3–62) [5,9,11].

## Epidemiological modelling

Let *f*_s_ denote the probability mass function of the serial interval of EVD, where the serial interval is defined as the time from illness onset in the primary case to time of illness onset in the secondary case [12]. Then *f*_s_, of length *s* days, is given by:

$$f_{s}=G\left(\text{s}\right)-G\left(\text{s}-1\right).$$

For *s* > 0, *G*(s) represents the cumulative distribution function of the gamma distribution. We characterised the expected number of new incident cases E[*c_i,t_*] in HZ *i* at symptom onset week *t* as follows:

$$\text{E}\left[c_{i,t}\right]=\underset{j}{\sum }r_{ij,t}\overset{\infty }{\underset{s=1}{\sum }}c_{j,t-s,}f_{s},$$

where *r*_ij_ denotes the average number of cases in HZ *i* infected by a single individual from HZ *j*. Here we assume that the incidence, *c*_i,t_, follows a Poisson sampling process with expected value E[*c*_i,t_].

The reproduction matrix for seven geographic HZs is given by:

$$M_{t}=\left(\begin{array}{cccc} \begin{array}{cccc} r_{11,t} & r_{12} & r_{13} & r_{14} \end{array} & r_{15} & r_{16} & r_{17}\\ \begin{array}{cccc} r_{21} & r_{22,t} & r_{23} & r_{24} \end{array} & r_{25} & r_{26} & r_{27}\\ \begin{array}{cccc} r_{31} & r_{32} & r_{33,t} & r_{34} \end{array} & r_{35} & r_{36} & r_{37}\\ \begin{array}{cccc} \begin{array}{c} r_{41}\\ r_{51}\\ r_{61}\\ r_{71} \end{array} & \begin{array}{c} r_{42}\\ r_{52}\\ r_{62}\\ r_{72} \end{array} & \begin{array}{c} r_{43}\\ r_{53}\\ r_{63}\\ r_{73} \end{array} & \begin{array}{c} r_{44,t}\\ r_{54}\\ r_{64}\\ r_{74} \end{array} \end{array} & \begin{array}{c} r_{45}\\ r_{55,t}\\ r_{65}\\ r_{75} \end{array} & \begin{array}{c} r_{46}\\ r_{56}\\ r_{66,t}\\ r_{76} \end{array} & \begin{array}{c} r_{47}\\ r_{57}\\ r_{67}\\ r_{77,t} \end{array} \end{array}\right).$$

This matrix is referred to as a next-generation matrix (NGM) in a fully susceptible population [13]. Using this matrix, we derive the instantaneous time-dependent effective reproduction number, *R*_t_, for the national transmission dynamics from the largest eigenvalue of the NGM. Under the assumption that the per-contact infection probability and the generation interval are consistent over time across HZs, the NGM quantifies the local (within zone) and inter-zone (across zones) patterns of transmission [14]. Then, the value of *R*_t_ for a specific HZ *j* is the sum of the local and inter-zone transmission (outgoing to other HZs), or the sum of column *j*.

Local transmission (within-zone) dominates the overall transmission dynamics, so we estimate these rates as time-dependent parameters. Inter-zone transmission also contributes to the generation of secondary cases, but to a comparably smaller degree; thus, we model it as an invariant quantity for simplicity. The serial interval is characterised using a gamma distribution with the mean and standard deviation (SD) at 15.3 and 9.1 days, respectively [15]. We fixed the maximum value of the serial interval at 6 weeks at 0.99, which is the cumulative probability of the gamma distribution at 6 weeks.

Using the model calibrated to the epidemic data and the latest estimates of the reproduction matrix, we generated a 4-week forecast assuming that the *R*_t_ remains stable throughout the period [16]. The forecast period is from week 39 to week 42 (30 September–27 October 2019). We estimated model parameters and made projections using a Monte Carlo Markov Chain (MCMC) method in a Bayesian framework. Point estimates and corresponding 95% credibility intervals (CrI) were drawn from the posterior probability distribution. All statistical analyses were done in R version 3.5.2 (R Foundation for Statistical Computing, Vienna, Austria) and the ‘rstan’ package (No-U-Turn-Sampler (NUTS)).

## Findings from the real-time outbreak analysis

**Figure 1:**
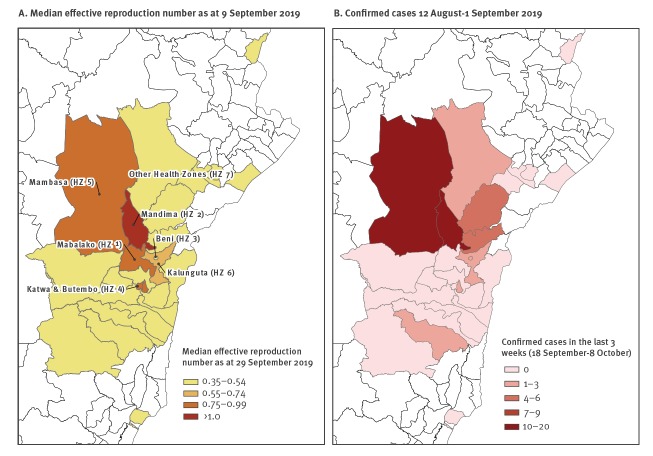
Geographical heterogeneity of Ebola virus disease reproduction number and confirmed cases across health zones, Democratic Republic of the Congo, 8 October 2019

Results indicate substantial spatial-temporal variation in *R*_t_ of Ebola virus across the seven HZs (Figure 1). The median effective reproduction number as of 8 October and the total number of new confirmed cases between 18 September–8 October 2019 by HZ are presented in Figure 1A and 1B, respectively.

**Figure 2:**
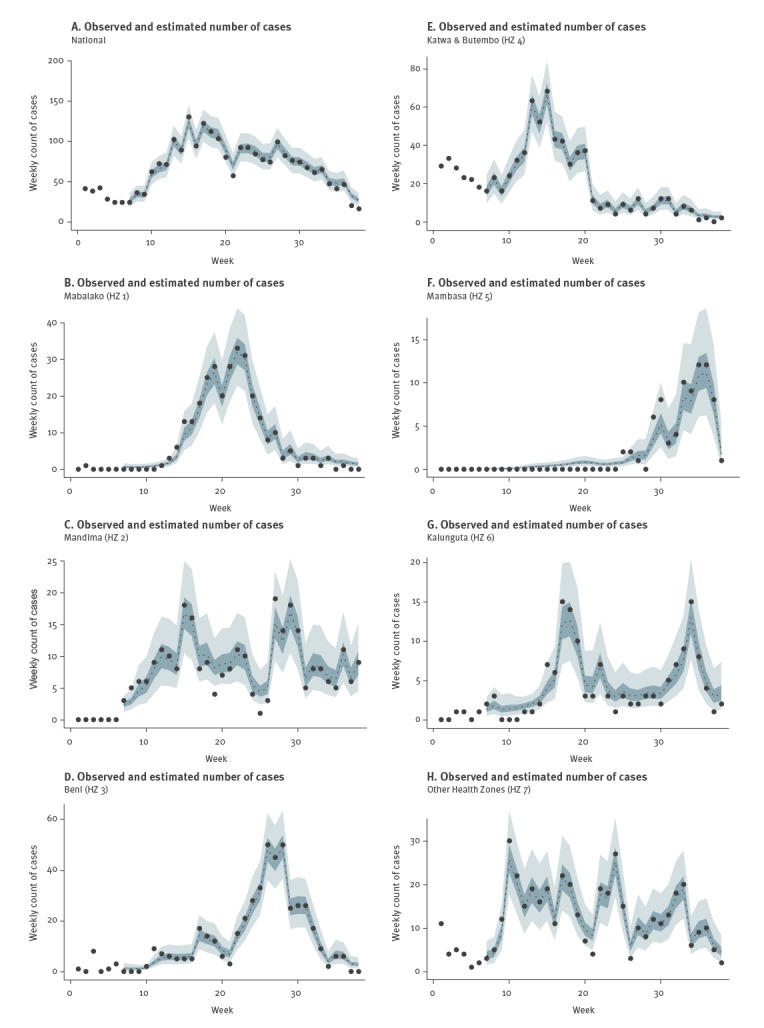
Observed and estimated number of Ebola virus disease cases by health zone, Democratic Republic of the Congo, January–September 2019 (n = 2,498)

We estimated the reporting delay adjusted EVD incidence for each week (*t*) at the national level and for each HZ from week 7 to week 38 (18 February–29 September 2019) (Figure 2) and the actual reported cases falls within the adjusted CrI for each HZ/national level.

HZ: health zone.

Light and dark indicates 95% and 50% credible intervals for posterior estimates, respectively.

**Figure 3:**
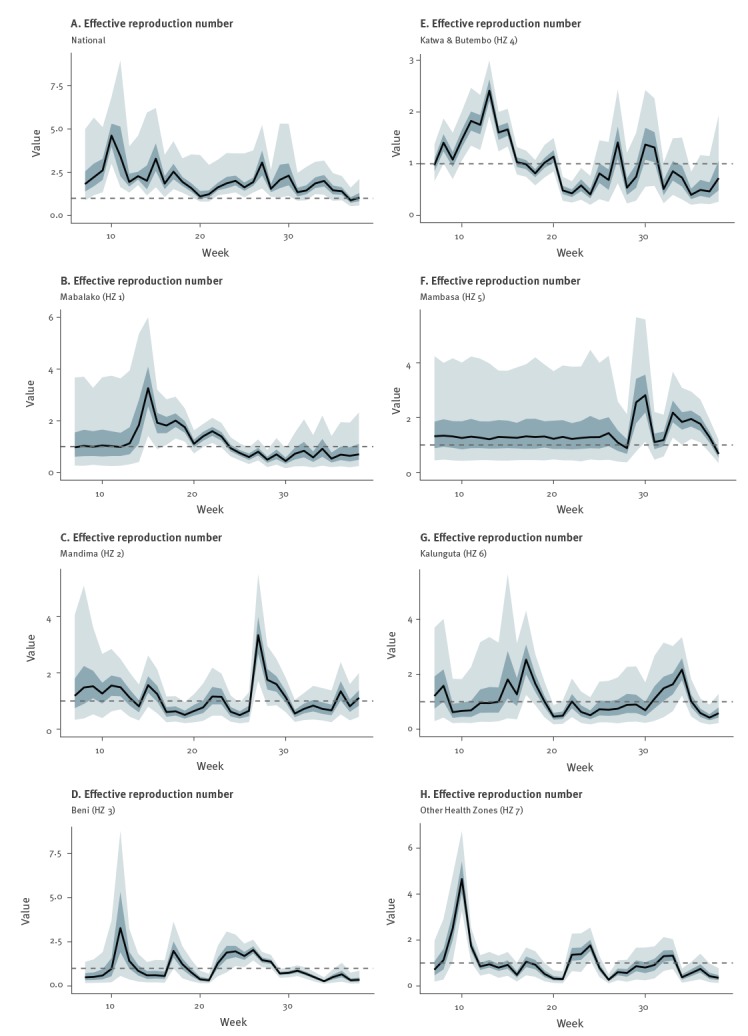
Time-dependent Ebola virus disease effective reproduction number by health zones, Democratic Republic of the Congo, January–September 2019

We derived weekly estimates of the effective reproduction number, *R*_t_, for the national and HZ levels (Figure 3). We found that the overall national *R*_t_ lies well above the epidemic threshold of 1.0 for most of 2019, with a multimodal pattern. After brief decline in July, our latest estimate of *R*_t_ at the national level is 1.03 (95% CrI: 0.59–2.12), indicating sustained transmission with substantial uncertainty straddling the epidemic threshold. Our estimates are also supported by the sensitivity analyses that examine the influence of small variations in the mean serial interval on our estimated *R*_t_ (Supplementary Figure S1).

HZ: Health Zone.

The national effective reproduction number were calculated from the dominant eigenvalue of next-generation matrix. Light and dark indicates 95% and 50% credible intervals for posterior estimates, respectively. Horizontal grey dashed line shows the reproduction number at 1.0 for reference, below which the epidemic goes to decline.

Week 1 on horizontal axis corresponds to 7 January 2019.

**Figure 4:**
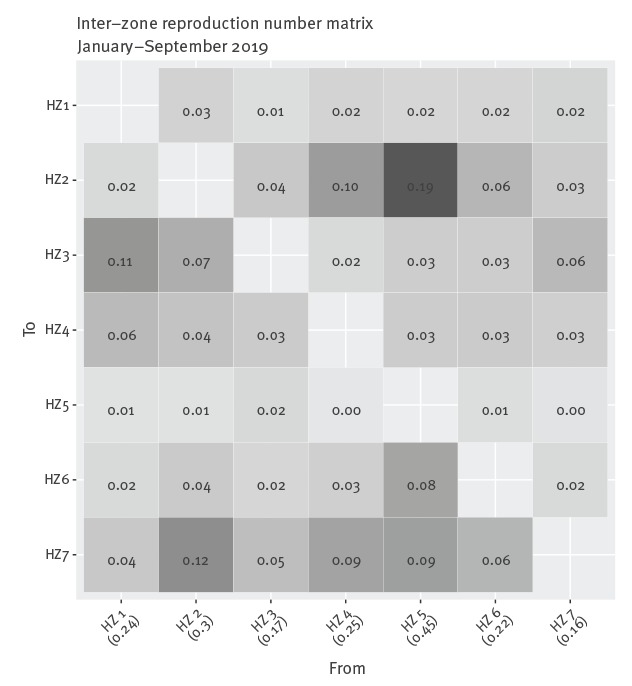
Inter-zone Ebola virus disease reproduction number matrix, Democratic Republic of the Congo, January–September 2019

Examining inter-zone transmission, where *r*_ij_ stands for the reproduction number from HZ *j* to HZ *i*, the median value is 0.03 (95% CrI: 0.00–0.12), with a maximum *r_ij_* value of 0.19 from Mambasa (HZ 5) to Mandima (HZ 2), and a minimum value of 0.00 from Katwa and Butembo (HZ 4) to Mambasa (HZ 5) (Figure 4). The sum of the outgoing *r*_ij_ values for a given HZ *j* yields the combined inter-regional reproduction number from HZ *j* to each of the other HZs. Mandima (HZ 2) and Mambasa (HZ 5) have notably higher outgoing transmission potential, with values of 0.30 and 0.45, respectively (Figure 4). Including local transmission (within-zone) using the latest estimate of *R*_t_, each column (or total *R_t_*) corresponding with its respective HZ increases to 0.70, 1.11, 0.35, 0.72, 0.67, 0.58 and 0.35 from HZ 1 to 7, respectively.

HZ: health zone.

HZ 1, 2, 3, 4, 5, 6 and 7 indicate Mabalak, Mandima, Beni, Katwa and Butembo, Kalunguta, and Other HZs, respectively. Number in the bottom line is the sum of the column, indicating the total transmission (within-zone and inter-zone transmission) by HZ.

Our short-term forecasts (week 39–week 42) are shown in Supplementary Figure S2. The predicted total number of cases from Mabalako (HZ 1) to Other HZ (HZ 7) is estimated to be 6.5 (95% CrI: 2.2–37.2), 31.2 (95% CrI: 11.3–96.6), 9.6 (95% CrI: 4.22–24.6), 11.4 (95% CrI: 4.0–47.4), 3.5 (95% CrI: 1.1–14.1), 8.5 (95% CrI: 3.5–26.7) and 14.0 (95% CrI: 6.5–30.1), respectively, while the predicted total number of cases across health zones is estimated at 95.2 (95% CrI: 53.0–185.2).

## Discussion

This article to assesses the EVD transmission potential at the national and HZ levels through the dynamics of *R*_t_ for the ongoing outbreak in the DRC, January–September 2019. Our national monthly level estimates indicate an overall decreasing trend in mean *R*_t_ from 2.18 in July 2019 to 1.20 in September 2019. The recent national *R*_t_ decline in July is consistent with changes in surveillance indicators, including a decline in the reporting delay from an average of 25.7 (CI: 17.4–36.04) days in February 2019 to 11.02 (CI: 10.28–11.8) days in September 2019. Furthermore, there has been an improvement in contact tracing with almost 90% of the contacts being followed daily in September 2019 associated with the recent case decline in the region [9,17,18].

Vaccination rates increased in the DRC by 47.9% from June 2019 to July 2019 among the people at risk for EVD, suggesting that public health measures have improved, supported by the gradual decline observed in EVD transmission. However, despite the improved control efforts, the frequency of violent attacks on healthcare centres and healthcare teams has remained consistent with an average of five attacks per month in 2019; possibly influencing disease persistence and suboptimal diagnostic delays [5,19]. Increased EVD transmission from February–March 2019 (week 7–11) and from mid-June to mid-July 2019 (week 24–28) may be associated with documented attacks to response efforts in Katwa and Butembo (HZ 4) and in North Khivu (Figure 3) [1]. This highlights the need to further strengthen the Ebola control efforts by improving the security situation in the affected health zones. This will have a positive impact on the infection control practices in the affected areas and enhance community engagement in order to extinguish all transmission chains.

Our findings support spatial heterogeneity in transmission, with recent *R*_t_ estimates ranging from 0.4 in Beni (HZ 3) to 1.1 Mandima (HZ 2). The transmission rates in Mandima (HZ 2), where the epidemic originated, suggests case reintroduction and exportations within and across the HZ as potential contributing factors to the ongoing epidemic [20,21]. On the other hand, public mistrust in the health authorities has contributed to case resurgences in Beni (HZ 3), a region that reported 30 Ebola community deaths in July 2019. There are signs of gradual improvement in control efforts including active contact tracing and vaccinations, but such efforts could be further enhanced [5].

Our study has several limitations. While we relate the observed fluctuations in *R*_t_ to outbreaks of violence, spatially-refined data would be required to explain the spatial variability in *R*_t_ over the course of the epidemic. Incorporating detailed epidemiological data (age, sex, etc.) as well as the timing, duration and intensity of public health efforts/disruptions into a mechanistic transmission model (or by conducting complementary spatial autoregressive modelling analyses) may allow the sources of spatial heterogeneity to be investigated [22]. We note that the inter-zone reproduction number for our analyses is taken as an 9-month average (January–September 2019) to facilitate the inference of the reproduction matrix. Further, Other HZ (HZ 7) in our analyses comprised of several HZs, for which we were not able to assess their transmission dynamics.

The *R*_t_ of the ongoing Ebola epidemic in DRC continues to display fluctuations with our most recent national estimate of *R_t_* reaching values slightly above the epidemic threshold of 1.0. Findings indicate that security incidents in the affected region continue to hamper the effectiveness of control interventions.

## Supplementary Data

647000 Supplement S1
