# Supplementary material for: Spatial variability in the reproduction number of Ebola virus disease, Democratic Republic of the Congo, January–September 2019
Source: Euro Surveill. 2019 Oct 17;24(42):1900588. doi: 10.2807/1560-7917.ES.2019.24.42.1900588 (PMC6807257; doi:10.2807/1560-7917.ES.2019.24.42.1900588)
Supplement: Supplement S1 [file 1900588_MITUMOTO_SupplementS1.pdf]

Supplement S1:

Transmission dynamics of Ebola virus disease, Democratic Republic of the Congo, January 2019 – September 2019

Mizumoto K, Tariq A, Roosa K, Kong J, Yan P, Chowell G

Disclaimer

This supplementary material is hosted by Eurosurveillance as supporting information alongside the article “Transmission dynamics of Ebola virus disease, Democratic Republic of the CongoDRC, January 2019 – September 2019” on behalf of the authors who remain responsible for the accuracy and appropriateness of the content. The same standards for ethics, copyright, attributions and permissions as for the article apply. Supplements are not edited by Eurosurveillance and the journal is not responsible for the maintenance of any links or email addresses provided therein.

1. Supplementary

2. Supplementary Results

3. Appendix Table S1

4. Appendix Figure S1, S2, S3

Supplementary

Reporting delay adjusted incidence

Because outbreak curves describing epidemic spread in near real time may be distorted by reporting delays, we adjusted the crude incidence rates for reporting delays using statistical methods. At the national level, for each case, the reporting delay is defined as the difference (in weeks) between the week of reporting and week of symptoms onset. We used a nonparametric method that adapts survival analysis for use with right truncated data, employing estimated reverse time hazards to adjust for reporting delays [1-3]. Then we incorporate the latest reporting periods to estimate the reporting delay probabilities and the random effects in the reporting delays to yield more robust and realistic prediction intervals. This method involves expressing the conditional reporting delay distribution as the product of conditional probabilities[3]

The crude incidence curve for each health zone was adjusted according to the national reporting delay adjustment factor to derive zone-level adjusted incidence curves. For these analyses, we focus on adjusted weekly incidence data for weeks in 2019 to better understand recent transmission dynamics; week 1 corresponds to 7 January 2019. Because the last reported week of incidence is well-known to be affected by downward bias, we exclude it from our analysis (e.g., lag of 1 week)[3] .

Methods for extracting the data from the figure

We have developed a new image processing method for extracting data from a bar chart with Matlab (R2018a) (Mathworks, Inc.). The algorithm requires users to select the origin, far end of x-, and y-axis as well as upper limits for the x and y axes. in a bar chart figure, respectively. After that, Then, the color figure is converted to the grayscale, where foreground information is separated from figure background by grayscale intensity. All bullet squares for legend are automatically detected by (1) difference between their widths and heights; and (2) absolute size in the resulting grayscale image. Colors of detected legend squares are used to identify bars for different disease areas. For each disease area in a figure, bars are detected by color information. The top edge of each bar is detected by convolving the resulting binary image with a 3x3 Prewitt filter. Lengths of all detected bar edge candidates are computed. All edge candidates with edge length either too long or too short are removed. The figure is partitioned into vertical regions by Time bins on the x-axis. Within each Time bin, the developed method scans from top to bottom until either a valid edge or the x-axis is reached. The y-coordinate of the most majority of edge pixels sharing the same y-coordinates is selected as the height of a bar within a given Time bin. If no valid edge is found, the y-coordinate of x-axis is taken for that Time bin. This analysis process is repeated over all Time bins along the x-axis. In the end, all extracted y-coordinates are normalized and mapped to their actual y-values.

Supplementary results

We estimated the reporting delay adjusted EVD incidence for each week (t) at the national level and for each health zone from week 7 to week 38 (18 February – 29 September, 2019) (Figure 2) and the actual reported cases falls within the adjusted credibility interval for each HZ/national. The total EVD cases for each of the health zones and at the national level are 298.9 (95% CrI: 267.1, 331.6), 294.2 (95% CrI: 263.3, 327.2), 461.3 (95% CrI: 420.5, 502.2), 647.8 (95% CrI: 600.7, 700.9), 80.6 (95%CrI: 65.2, 97.9), 156.5 (95%CrI: 134.4, 180.3) and 435.6 (95% CrI: 397.8, 477.0) from Mabalako (HZ 1) to Other Health Zones (HZ 7) and 2376.5 (95% CrI: 2283.9, 2470.6) at the national level. In contrast, the reporting delay adjusted actual EVD cases during corresponding study period are 291, 280, 450, 634, 78, 144, 424 and 2301, respectively. Percentage coverage of the 95%CrI of estimated data for observed data is 94% (30/32) for the national, and 66% (21/32), 91% (29/32), 75% (24/32), 94% (30/32), 38% (12/32), 81% (26/32) and 91% (29/32) from Mabalako (HZ 1) to Other Health Zones (HZ 7), respectively.

Our latest estimate of R_t at the national level is 1.03 (95% CrI: 0.59, 2.12), with 55% of R_t estimates lying above the epidemic threshold of 1.0. Mabalako (HZ 1), Mandima (HZ 2), Beni (HZ 3), Katwa & Butembo (HZ 4), Mambasa (HZ 5) and Kaluguta (HZ 6) and Other Health Zones (HZ 7) also have total (within-zone and inter-zone) Rt values above the epidemic threshold, with percentages of 29%, 61% 1%, 29%, 8%, 11% and 1% respectively (Table S1). Ignoring the inter-zone transmission and looking only locally, or within-zone, Mabalako (HZ 1), Mandima

(HZ 2), Katwa & Butembo (HZ 4) and Kaluguta (HZ6) still exhibit a potential for sustained transmission, with 17%, 25%, 15% and 2% of the corresponding density of R_t lying at values greater than 1, respectively (Figure S2).

Tables

Table S1 – The latest estimate of median effective reproduction number and fraction of the density of R above the threshold of 1.0.

		Range	Percentage over 1.0
National		1.03 (95%CrI§: 0.59, 2.12)	55%
HZ! 1, Mabalako	Total ‡	0.70 (95%CrI: 0.24, 2.33)	29%
	Local¶	0.45 (95%CrI:0.02, 2.04)	17%
HZ 2, Mandima	Total	1.11 (95%CrI: 0.44, 1.99)	61%
	Local	0.72 (95%CrI: 0.12, 1.59)	25%
HZ 3, Beni	Total	0.35 (95%CrI: 0.14, 0.87)	1%
	Local	0.14 (95%CrI: 0.01, 0.68)	0%
HZ 4, Katwa & Butembo	Total	0.72 (95%CrI: 0.27, 1.94)	29%
	Local	0.47 (95%CrI: 0.02, 1.68)	15%
HZ 5, Mambasa,	Total	0.67 (95%CrI: 0.34, 1.18)	8%
	Local	0.13 (95%CrI: 0.01, 0.48)	0%
HZ 6, Kalunguta	Total	0.58 (95%CrI: 0.23, 1.29)	11%
	Local	0.25 (95%CrI: 0.01, 0.90)	2%
HZ 7, Other Health Zones	Total	0.35 (95%CrI: 0.13, 0.78)	1%
	Local	0.14 (95%CrI: 0.01, 0.53)	0%

§CrI: 95% credibility intervals (CrI)

! HZ: Health zone

‡Total transmission includes within-zone and inter-zone transmission

¶Local includes within-zone transmission only

Figure S1. Sensitivity analysis of the Ebola virus disease effective reproduction number, Democratic Republic of the Congo, January - September 2019.

Result of sensitivity analysis is shown. Medians of the effective reproduction number are presented assuming a mean serial interval ranging from 13.3 to 17.3. Full line represents median of posterior estimates derived from the mean serial interval of 15.3 days, respectively, while area surrounded by grey color corresponds to median of posterior estimates derived from the mean serial interval of 13.3 and 17.3 days. The horizontal grey dashed line indicates the reproduction number at 1.0 for reference, below which the epidemic follows a declining trend. Week 1 on horizontal axis corresponds to 7 January, 2019. HZ is the abbreviation of Health Zone.

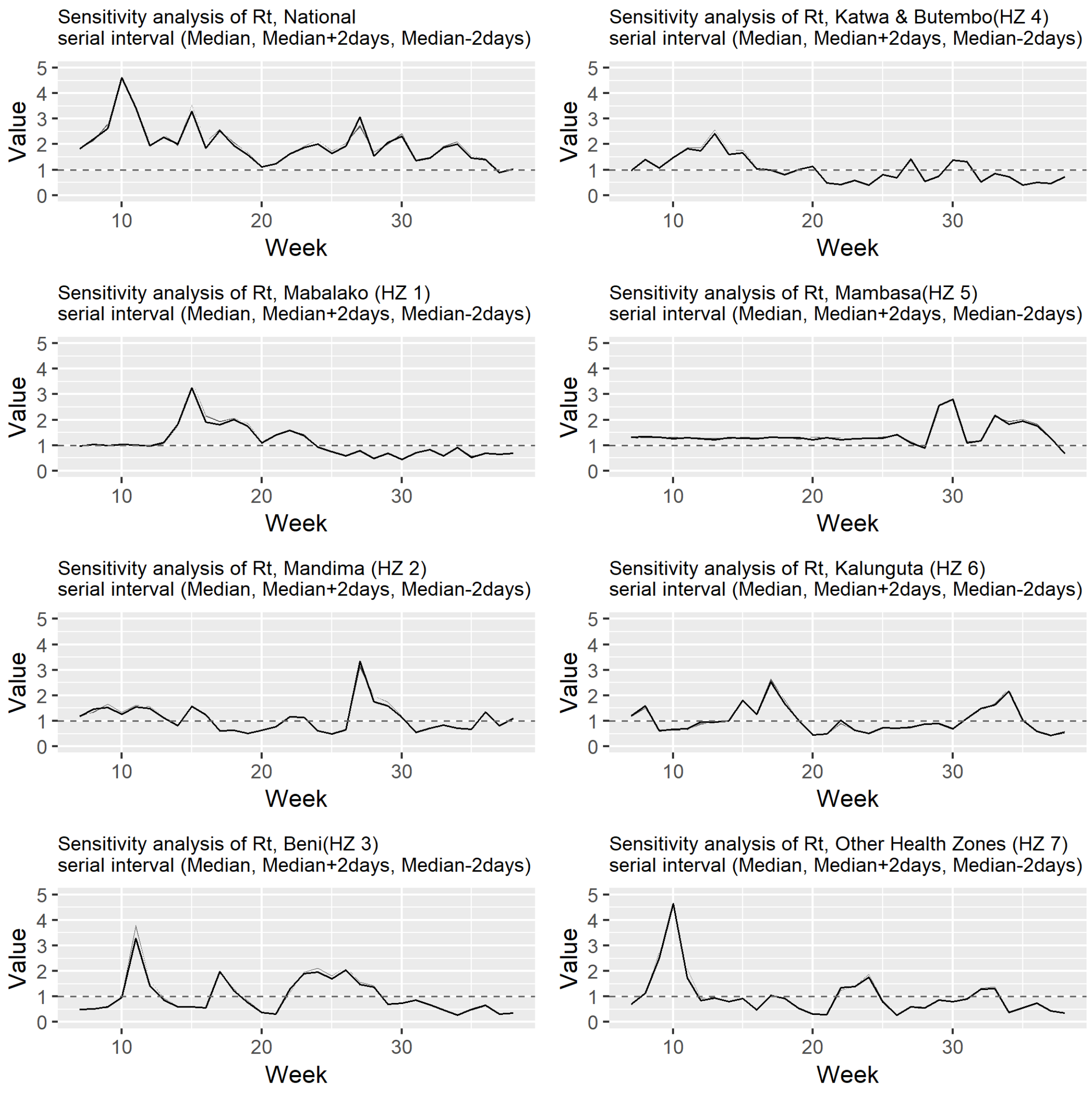

Figure S2. Observed and projected Ebola virus disease incidence of illness onset over time by health zones, Democratic Republic of the Congo.

The dots shows the observed incidence over time. The dashed black line indicate 50% quantiles, while light and dark indicates 95% and 50% credible intervals of the projected incidence. The vertical dotted lines depict the time of projection. Week 1 on horizontal axis corresponds to 7 January, 2019. HZ is the abbreviation of Health Zone.

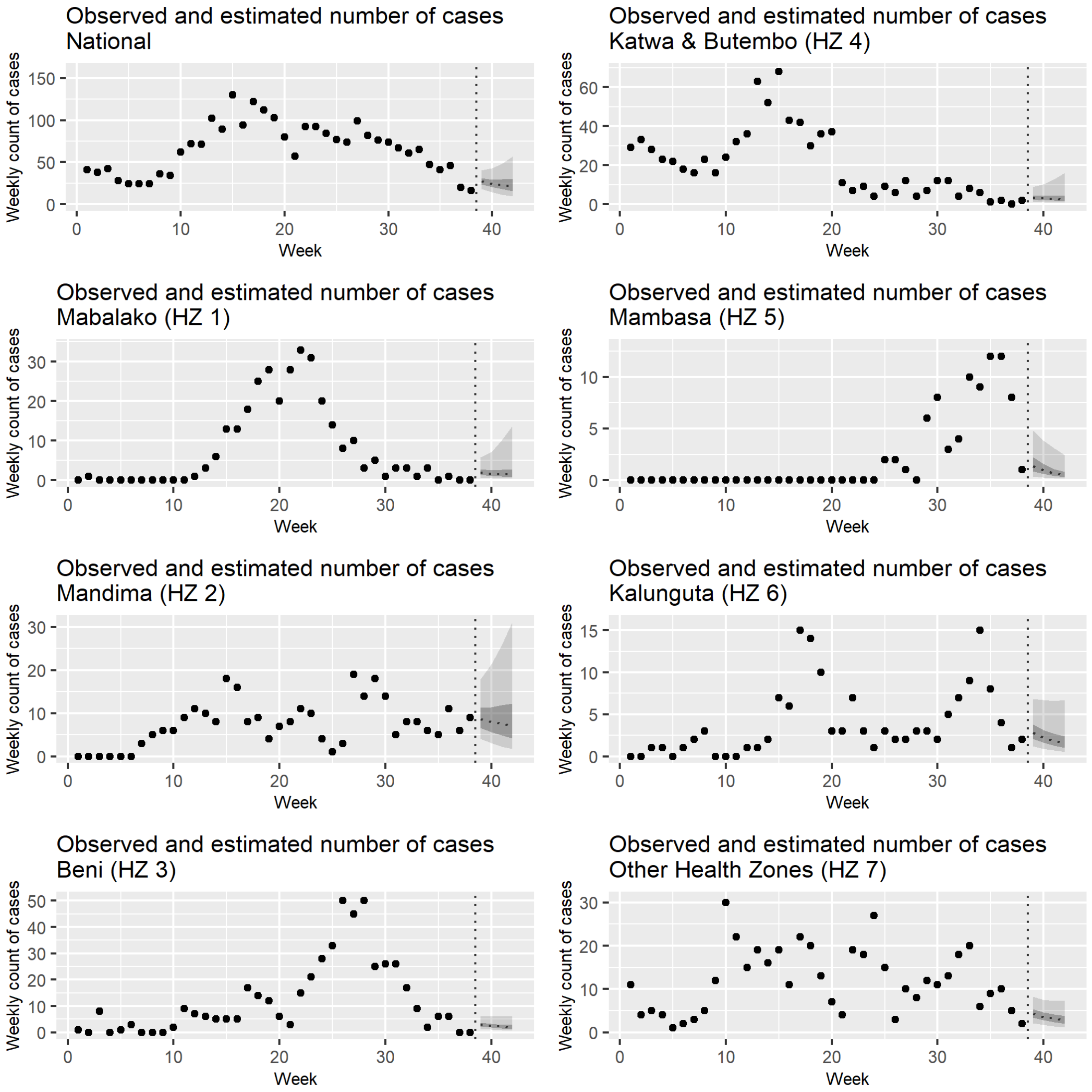

Figure S3. Ebola virus disease reproduction number distribution for national, health zone (total, local) and inter-zone, Democratic Republic of the Congo, January - September 2019.

(A) National and total transmission (within-zone and inter-zone transmission) by health zone, (B) Local transmission (within-zone only) by health zone, (C) Inter-zone transmission by health zone. A and B present the latest estimate of effective reproduction number. HZ is the abbreviation of Health Zone.

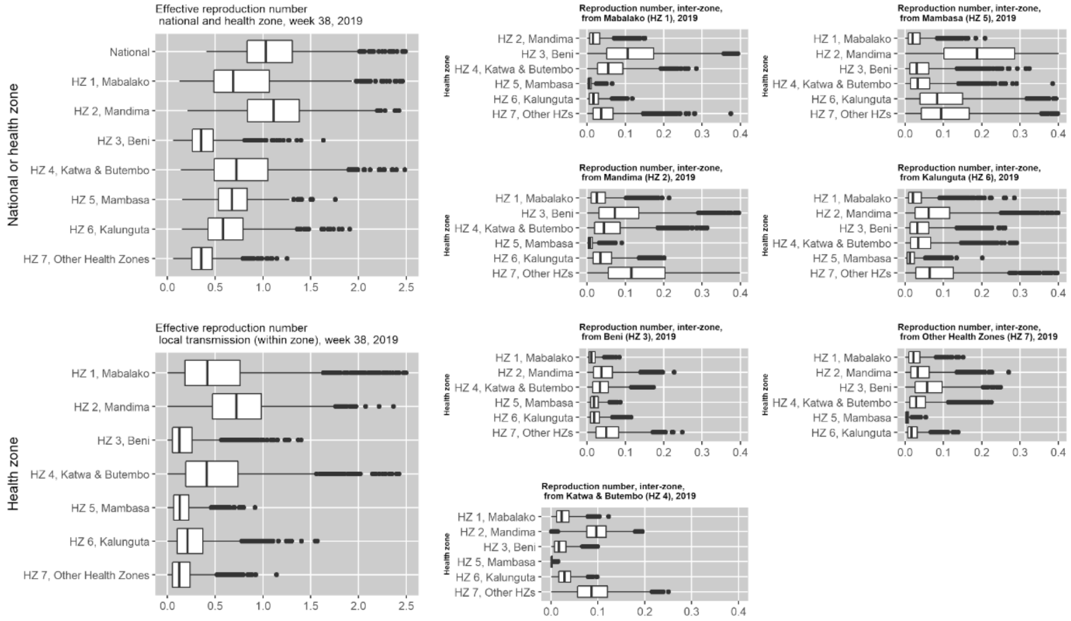

REFERENCES

1. Chowell, G. and H. Nishiura, Transmission dynamics and control of Ebola virus disease (EVD): a review. BMC Medicine, 2014. 12: p. 196-196.
2. Diekmann, O., J.A.P. Heesterbeek, and M.G. Roberts, The construction of next-generation matrices for compartmental epidemic models. 2010. p. 873-885.
3. Nishiura, H. and G. Chowell, Early transmission dynamics of Ebola virus disease (EVD), West Africa, March to August 2014. Euro Surveill, 2014. 19(36).
